# Single-Cell RNA-Seq Profiling of Transposable Element Expression in Human Peripheral Blood Cells During Viral Infections

**DOI:** 10.3390/ijms27031286

**Published:** 2026-01-28

**Authors:** Oleg D. Fateev, Vasily E. Akimov, Olga V. Glushkova, Aleksandr V. Bolbat, Azat V. Abdullatypov, Olga A. Antonova, Vladimir V. Shiryagin, Nikolai A. Bugaev-Makarovsky, Vladimir S. Yudin, Anton A. Keskinov, Sergei M. Yudin, Dmitriy V. Svetlichny, Veronika I. Skvortsova

**Affiliations:** 1 Federal State Budgetary Institution “Center for Strategic Planning and Management of Biomedical Health Risks” of the Federal Medical-Biological Agency (FSBI “CSP” of FMBA of Russia), Pogodinskaya str. 10, bld. 1, Moscow 119435, Russia; 2 Federal Medical-Biological Agency (FMBA of Russia), Volokolamskoye Shosse, 30, Moscow 119121, Russia

**Keywords:** transposable elements, viral infection, influenza A, HIV, COVID-19, scRNA-seq

## Abstract

Transposable elements (TEs) are key regulators of immunity in both health and disease. It has been proven that the activity and transcriptional expression levels of TEs increase during viral infections, correlating with the antiviral response. This study investigates non-LTR TE (LINE, SINE, and SVA) transcriptomic signatures in human PBMCs during infections caused by influenza A virus, HIV, and SARS-CoV-2 (Delta/Omicron variants) using single-cell RNA sequencing (scRNA-seq) data from 98 patients. In the HIV and SARS-CoV-2 patient cohorts, unique cell-specific TE expression patterns were identified that allow for the differentiation of disease severity, prediction of disease progression, and assessment of the therapy’s efficacy. The expression of LINE elements was found to be more dependent on the nature and course of the disease than that of SINE elements. The most variable TE expression profile was observed in precursor cytotoxic T-lymphocytes (T CD8+ Naive cells), which depended on the virus type and the severity of the viral disease. For this cell type, a bioinformatic analysis of the co-expression regulation of TE transcriptional networks and transcription factors during viral infections was performed. This analysis identified key players among those most involved in virus-specific responses, which could serve as diagnostic biomarkers or therapeutic targets for treating diseases caused by influenza A virus, HIV, and SARS-CoV-2. This work confirms the involvement of non-LTR TEs in mediating antiviral responses. Further research into the mechanisms of TE participation in antiviral defense is necessary to recommend them as potential biomarkers for the diagnosis, monitoring, and assessment of antiviral therapy, or as therapeutic targets for viral infections of various origins.

## 1. Introduction

One of the primary sources of structural variability in the human genome is the activity of transposable elements (TEs). TEs are DNA repeat sequences found in all eukaryotic genomes, ranging from 50 base pairs in length [1,2] and capable of moving (transposing) or self-copying within the genome. They occupy approximately 45–50% of the human genome [3]. TEs are divided into two classes—retrotransposons and DNA transposons—which differ in their transposition mechanisms and activity [4]. The more extensive class of TEs is retrotransposons, which transpose via a “copy-and-paste” mechanism mediated by the transcription of DNA into RNA, followed by reverse transcription and insertion into a new genomic site primed by a target. Only some retrotransposons are capable of actively inserting into the human genome. The class of human retrotransposons includes non-active in the human genome LTR elements, which have long terminal repeats and encode a full set of elements necessary for their autonomous replication in the genome, and non-LTR elements—autonomous LINE (the most numerous being the L1 family), SINE (the most common representatives being Alu), and SVA (SINE-VNTR-Alu). In the human genome, three families of non-LTR retrotransposons—Alu, L1, and SVA—remain transcriptionally active and continue to stimulate the insertion of new mobile elements. At present, only L1 is known as an active and autonomous retrotransposon, providing the retrotransposition of non-autonomous SINE and SVA [5,6]. Their retrotransposition largely depends on the existence of functional and active L1 sequences [7,8,9]. Collectively, L1, Alu, and SVA sequences account for nearly 30% of the human genome, with approximately 500,000 L1 sequences, 1,100,000 Alu sequences, and 3000 SVA sequences identified [4,10]. The area of interest in this study was to investigate the activity of non-LTR transposon families, some members of which have retained the ability to retrotranspose in the human genome. TE insertion sites are cell-specific and depend on multiple factors, such as selective pressure, drift, recombination rate, ploidy level, genomic background, locus-specific chromatin accessibility, and transcriptional activity [11,12]. Once integrated into a new genomic location, retrotransposons remain there and, in most cases, lose their activity. TEs of the other class—DNA transposons—move via a “cut-and-paste” mechanism. These are DNA elements whose translocation is facilitated by transposase enzymes. Such elements constitute about 3% of the human genome, but no active TEs from this class are known to exist today [13]. Nevertheless, DNA transposons have played a significant role in the evolution of the human genome [14]. Active TEs can integrate into both non-coding and coding regions of the genome. The effect of TE insertions into new sites can lead to the insertional mutagenesis of key genes, affecting fundamental biological processes such as genome stability, enhancer activity, RNA splicing, and innate immunity [15]. Dormant TEs, which have lost their ability to transpose, are suppressed under normal conditions in somatic cells by a complex, multi-level regulatory system. However, they can be reactivated by cellular stress or epigenetic changes, leading to the production of virus-like genetic material [16]. The dysregulation of TEs leads to impaired function of cells, organs, and the organism as a whole, contributing to aging and age-related diseases [17]. TEs influence the organism’s phenotype not only through genomic insertions; they are a major source of non-coding RNAs (ncRNAs), which have a broad spectrum of regulatory effects, and facilitate the recruitment of transcription factors by serving as a source of tissue-specific enhancer, silencer, or promoter sequences [18,19].

TEs both act as key genome architects, driving evolutionary innovation, and make a substantial contribution to the pathogenesis of a wide range of diseases. On the one hand, TEs are involved in physiological processes such as embryonic development and the formation of immune responses. On the other hand, they mediate disturbances in nervous system development, stem cell differentiation, aging, immune pathologies, and cancer progression [20,21,22,23,24,25]. In humans, an imbalance in TE expression occurs in most pathological conditions. This is linked to the diversity of functions performed by TEs, including their ability to regulate innate and adaptive immunity, as well as the pathogenesis of autoimmune diseases, and the activation, differentiation, and tissue adaptation of T-lymphocytes [26]. TEs represent potential diagnostic biomarkers and therapeutic targets due to their disease-specific activation patterns and immunogenic properties. In this context, the study of TE biology is a new and promising area of pathoimmunology [27], including in the investigation of host responses during infectious diseases of viral origin that are accompanied by impaired immune reactivity.

It is known that transcriptional, expressional, or immunogenic disturbances in TE characteristics are observed in patients with infections caused by HIV-1, which directly destroys CD4 T-lymphocytes leading to the subsequent development of Acquired Immunodeficiency Syndrome [28]; SARS-CoV-2, which causes hyperstimulation of the immune system resulting in subsequent secondary immunodeficiency [27,29,30]; and influenza A virus, which leads to the death of immune cells and the development of a wide spectrum of immunodeficiencies [31,32].

The aim of this work was to identify cell-type-specific transcriptomic signatures of non-LTR transposable elements in human immune cells during infectious diseases of varying severity caused by influenza A virus, HIV-1, and SARS-CoV-2 (Delta or Omicron variants). To achieve this, we utilized scRNA-seq data profiling the transcriptome of PBMCs obtained from 98 individuals. Furthermore, using bioinformatic analysis, we assessed the co-expression regulation of transcriptional networks of TE and transcription factors (TFs) during these viral diseases.

## 2. Results

### 2.1. Single-Cell RNA Sequencing for Investigating TE Expression During RNA Viral Infections: PBMC Annotation and Clustering

**Figure 1:**
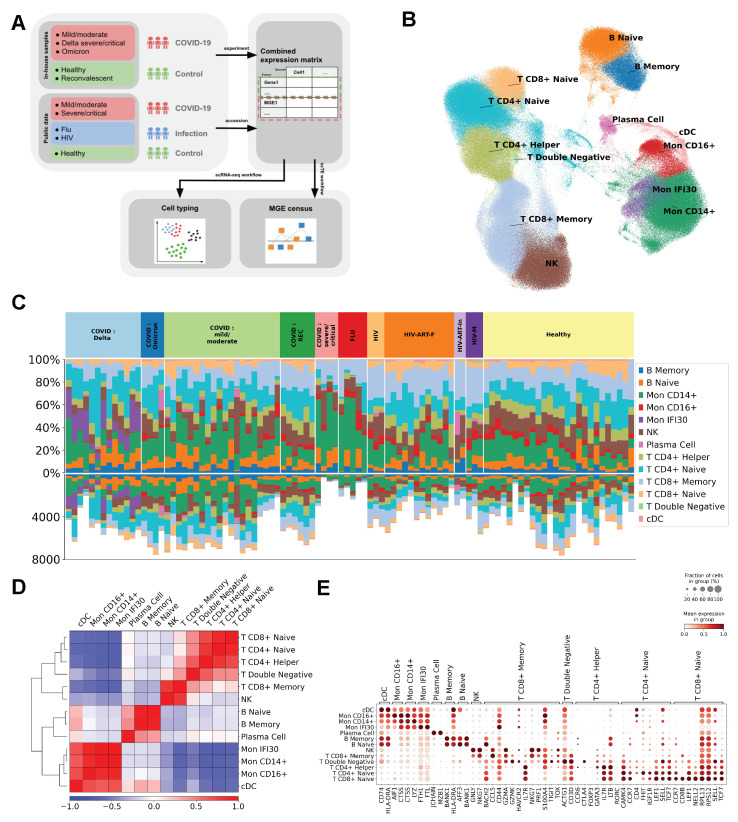
Single-cell transcriptomic profiling and annotation of PBMCs from patients with viral infections. (**A**) Study design schematic: merging in-house and public cohorts for comparative single-cell transcriptomics. (**B**) UMAP visualization of clustering based on gene expression profiles, showing annotated cell types. (**C**) Percentage and absolute count of each cell type in the analyzed samples. (**D**) Correlation matrix (Wilcoxon test) of gene expression profiles across different cell types. (**E**) Expression levels of known marker genes (according to the literature data) in the identified cell populations.

To study TE expression during RNA viral infections, we analyzed scRNA-seq results from peripheral blood samples of 72 patients with viral infections and 26 healthy patients. The study investigated PBMC samples from patients infected with influenza A virus, HIV-1, or SARS-CoV-2 (Delta and Omicron variants, varying disease severity) (Table 1, Figure 1A). Based on the scRNA-seq data, a combined matrix was generated to determine both TE activity and the cellular transcriptomic profile after annotation and subsequent clustering of cell types.

Given the different functional roles of various myeloid and lymphoid blood cell subtypes, the first stage of the research involved high-precision cell-type identification using the CellTypist tool [33]. To minimize batch effects and ensure an accurate comparative assessment of TE expression activity, only data from the 10× 3′ technology were used in this study. Five T-cell types were identified (T CD8+ Naive, T CD4+ Naive, T CD4+ Helper, T Double-Negative, T CD8+ Memory), three monocyte clusters (Mon IFI30, Mon CD16+, Mon CD14+), three B-lymphocyte subtypes (B Naive, B Memory, and Plasma Cells), as well as fractions of Natural Killer (NK) cells and conventional Dendritic Cells (cDCs) (Figure 1B and Figures S2 and S3). The results indicate typological proximity on the UMAP for phenotypically similar cell populations. Furthermore, high variability in relative cellular composition was detected not only between cohorts but also within groups (Figure 1C). This could be associated with differences in sample preparation protocols, individual variability, and the presence of cell types specific to particular conditions (for instance, the monocyte subtype Mon IFI30 is characteristic of SARS-CoV-2-Delta variant infection [34] and was not identified in any other samples). Correlation analysis of expression profiles revealed the clear separation of lymphoid and myeloid cells into distinct clusters, indicating the high-quality integration of heterogeneous single-cell expression data. Analysis of marker genes, known from the literature sources, We confirm. that their expression corresponded to the annotated cell types [33] (Figure 1D,E). For example, dendritic cells showed expression of the surface markers *CD74* and *HLA-DRA* Expression of the gene *CTSS* (Cathepsin S)-a characteristic monocyte marker-was detected in all three monocyte clusters, while CD16+ monocytes exhibited expression of the allograft inflammatory factor *AIF1*, and CD14+ monocytes showed expression of lysozyme (*LYZ*). Mature plasma cells were characterized by expression of the Immunoglobulin J Chain (*JCHAIN*) gene and *MZB1*, which encodes a protein specific to marginal zone B and B1 cells [33,35]. B-cells typically expressed the B-cell scaffold protein with ankyrin repeats gene *BANK1, HLA-DRA* (for memory B-cells), and *AFF* (for naive B-lymphocytes). *FTH1* and *FTL* helped identify IFI30 monocytes, a unique cell subtype found exclusively in the cohort of patients infected with the SARS-CoV-2-Delta variant [34]. The obtained cell type annotations were used for subsequent analysis of TE expression profiles within these same cell types.

To evaluate potential batch effects and overall consistency across samples, we performed correlation analyses using two distinct expression matrices: (1) the top 3000 highly variable protein-coding genes and (2) TE expression profiles. The Pearson correlation matrices for these two modalities are shown in Figure S1A and S1B, respectively. Across both representations, samples exhibit high within-group correlations (mean = 0.9302), which are significantly greater than between-group correlations (mean = 0.9111; difference = 0.0191; Mann–Whitney U test, *p* = $1.98\times 10^{-56}$), indicating strong reproducibility within biological conditions while preserving meaningful inter-group differences. To further assess integration quality, we performed a PCA based on the 3000 highly variable genes (Figure S1C,D). When colored by the experimental group (Figure S1C), samples largely intermingled, with the notable exception of samples from individuals infected with the SARS-CoV-2 Delta variant, which showed mild but discernible clustering-likely reflecting profound immune perturbations associated with this lineage. In contrast, coloring by dataset of origin (Figure S1D) revealed no pronounced clustering by source dataset, suggesting minimal technical batch effects. Together, these results demonstrate that our integrated dataset is robust, with high inter-sample concordance, and suitable for downstream joint analysis of both gene and TE expression.

Thus, the analysis of marker gene expression demonstrated the clear separation of PBMCs into cell subtypes. This indicates high sample quality, an adequate sequencing procedure, and proper data processing, thereby enabling further molecular genetic analysis of the transcriptome and TE activity.

### 2.2. Variations in Whole Blood Transcriptomic TE Signatures Depending on the Type of Viral Infection

**Figure 2:**
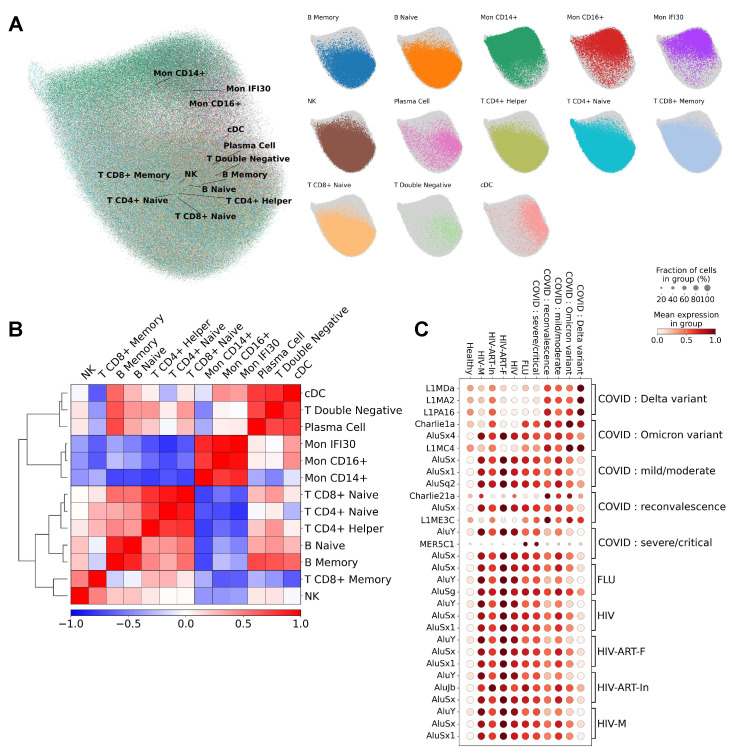
Landscape of TE expression in PBMCs during viral infections. (**A**) UMAP visualization of clustering based on TE expression with assigned cell type annotations. (**B**) Correlation matrix (Wilcoxon test) of TE expression profiles across different annotated cell populations. (**C**) Expression levels of TEs specific to certain conditions, compared across different cohorts.

The advantages and power of scRNA-seq lie in its ability to detect different cell types, their subpopulations, and unique cellular profiles characteristic of various diseases. Using these data, the preliminary annotation of PBMC cell types, and the RepeatMasker tool, we performed clustering of TEs based on cell types (Figure 2A). To assess the correlation between TE expression and cell subtype, we selected cell types based on marker gene expression, chose TEs with variable expression, performed dimensionality reduction and data clustering, and subsequently obtained a UMAP representation. While clear visual separation of clusters, compared to protein-coding genes, was not observed, cell-specific TE expression was identified in the context of gene groups (Figure 2A). The performed clustering revealed spatial separation of cells based on TE expression into myeloid and lymphoid lineages. The heatmap of TE expression correlation (Figure 2B) demonstrates similarity in expression profiles for phenotypically related cell types.

The question of specific expression of various TEs in the context of the studied patient cohorts with viral infections was investigated in more detail. For this purpose, the most differentially expressed (DE) TE were identified for each study group (Figure 2C). The following features of TE expression in PBMCs of patients with viral infections are noteworthy. First, all immune cells from cohorts with viral infections, as well as recovered individuals, had significantly higher expression levels of various TE types compared to the “Healthy” cohort. Second, patients from the influenza A and HIV cohorts were characterized by the highest expression of Alu family retrotransposons (SINE elements), while immune cells from the “COVID: Delta variant” cohort were distinguished by the high expression of L1 family representatives (LINE elements). Interestingly, the most specific TE expression signature was found for the “COVID: Delta variant” cohort-the highest level of differential expression was shown for TEs of the LINE1 retrotransposon family: L1MDA, L1MA2, and L1PA16. Furthermore, patients in the “SARS-CoV-2 Delta” cohort exhibited reduced levels of Alu family TEs and DNA transposon expression. Thus, our work reveals a new specific signature in the expression profile of immune cells from the “SARS-CoV-2-Delta” group, which warrants further study. Immune cells from the “COVID: Omicron variant” cohort were also characterized by high expression levels of L1MDA, L1MA2, and L1PA16 compared to all studied cohorts except “COVID: Delta variant”. It is also important to mention the virus-dependent specificity of DE DNA transposons. In our work, we identified three such DE TEs-Charlie1a, Charlie21a, and MER5C1-belonging to the hAT superfamily of DNA transposons [13]. Pronounced expression of Charlie1a was found in the “FLU” cohort and most groups of patients with coronavirus infection; Charlie21a is a DE TE in coronavirus infection, and MER5C1 is specific to the “COVID: severe/critical” and “FLU” cohorts.

Interestingly, immune cells from the HIV patient cohorts (“HIV-M” and “HIV-ART-F”) exhibited TE expression profile signatures that were indistinguishable before and after ART. These signatures were characterized by highly expressed SINE element TEs (Alu family) and minimal expression of DNA transposon and L1 TEs (L1PA16, L1MA2, L1MDA, L1MC4). However, upon completion of ART, the expression level of SINE elements in patient blood cells was the highest compared to other HIV cohorts. Severe HIV disease progression was accompanied by increased expression of Charlie21a. The expression profile of the “HIV-ART-In” cohort (patients with partially ART-suppressed infection) was characterized by a lower, yet statistically significant compared to the healthy cohort, differential expression of a broader spectrum of TEs (Figure 2A): all TEs represented on the heatmap (members of the L1, Alu retrotransposon families, and Charlie1a) were highly expressed, with the exception of Charlie21a and MER5C1.

Thus, unique TE expression signatures were identified that allow for differentiation of the acute phase of SARS-CoV-2 Delta variant infection (the “COVID: Delta variant” cohort) and distinction of completed ART (the “HIV-ART-In” cohort) compared to other HIV cohorts. These transcriptional signatures were characteristic of all cell populations within these cohorts. Furthermore, the consistency of TE transcriptional changes within each cohort was demonstrated using T CD8+ Naive cells (Figures S4 and S5).

### 2.3. Identification of PBMC Cell Types with Unique Virus-Specific Signatures of Differentially Expressed LINE and SINE Retrotransposons

**Figure 3:**
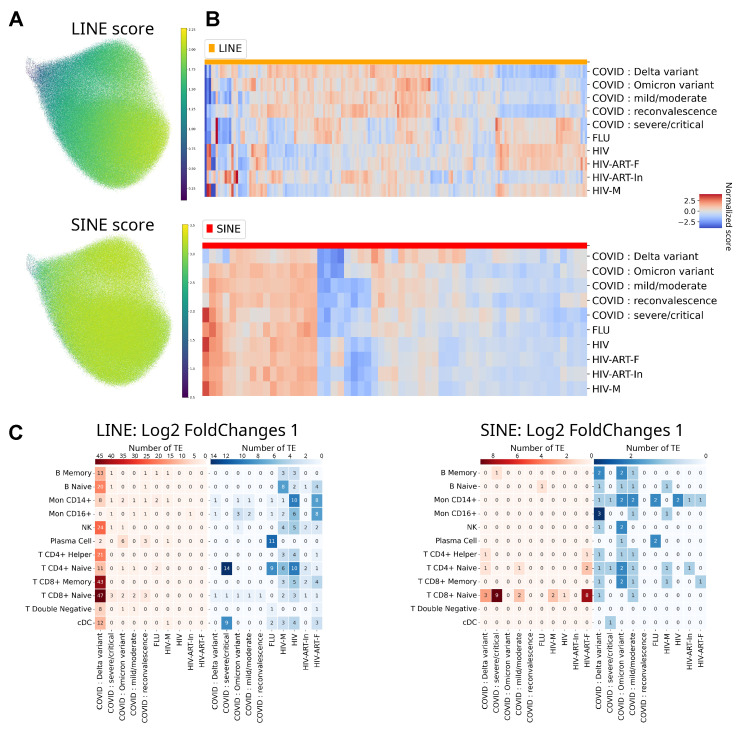
Cell-type and cohort-specific dynamics of LINE and SINE retrotransposon expression. (**A**) Distribution and expression level of LINE elements in blood cells, where the color scale reflects the normalized expression score. (**B**) Distribution and expression level of SINE elements in blood cells, where the color scale reflects the normalized expression score. (**C**) Quantitative summary of TE dysregulation across cell types and infections. The number of significantly upregulated (red) or downregulated (blue) LINE and SINE retrotransposons is shown for each PBMC subtype and patient cohort. Counts were obtained from single-cell differential expression testing (each cohort vs. healthy donors) within annotated cell clusters, preserving single-cell resolution in the statistical model.

Given that the majority of DE TEs between cohorts belonged to the LINE or SINE families, we analyzed the quantitative content of retrotransposons from these families across different cell types in all experimental cohorts. To investigate TE expression patterns characteristic of different viral infections, their expression levels in all cohorts were compared to those in healthy control samples (Figure 3A,B). The presence of DE TE classes was revealed, which were characteristic of most cohorts as well as specific to certain conditions. The expression profile of LINE family elements in HIV patients stands out, representing a distinctive feature of the blood immune cell transcriptional response to HIV infection. Furthermore, we analyzed the quantitative characteristics of DE for individual members of the LINE and SINE families across different cell types in the experimental cohorts relative to healthy donors; Log2 FC data are presented in Figure 3C. As shown in the figure, differential expression of LINE and SINE elements was identified depending on the nature of the viral infection. For instance, in cohorts with HIV infection (cohorts “HIV”, “HIV-M”, “HIV-ART-In”, “HIV-ART-F”), a significant decrease in LINE element expression was observed, particularly in CD4+ immune cells, which are known to be selectively targeted by HIV, and in monocytes. CD16+ monocytes in HIV infection cohorts also showed reduced LINE element expression. ART significantly alters the retrotransposon expression profile in PBMCs of HIV patients: in the “HIV-ART-In” and “HIV-ART-F” cohorts, the number of DE LINE elements with reduced expression is significantly lower across all cell types compared to the “HIV” and “HIV-M” cohorts. Additionally, an increase in the expression of a number of SINE elements is observed in naive CD4+ and CD8+ T-cells of HIV patients after ART compared to the “HIV” and “HIV-M” cohorts.

The “COVID: Delta variant” cohort exhibited a high expression level of LINE elements in all immune cells except for CD16+ monocytes, which were absent in patients of this group (Figure 1C and Figure 2C). These same CD16+ monocytes were characterized by a significant increase in the number of low-expression LINE elements.

The FLU patient cohort showed minor changes in the differential expression of the studied retrotransposons across all cell types. It is possible that TEs from the DNA transposon group are more involved in mediating cellular responses to this virus (Figure 2C). For this type of viral infection, the most variable changes in TE expression were observed in plasma cells, which typically exhibit high expression stability during antiviral responses to other viruses. This is likely related to specific complexities in the transcriptional programming of different antigen-specific plasma cell populations during influenza A infection [36].

Thus, PBMC cell types exhibiting divergent expression signatures of differentially expressed retrotransposons in response to different types of viral infections (influenza A, HIV, SARS-CoV-2) were identified. The most significant cell-specific differential changes in the quantitative level of differentially expressed retrotransposons across different experimental cohorts were found in naive T-lymphocytes, particularly in T CD8+ Naive cells, which are precursors to effector cytotoxic T-lymphocytes, playing a crucial role in antiviral immunity. Double-negative T-lymphocytes were characterized by the most stable expression levels of LINE and SINE elements, which showed little dependence on the type of viral infection.

### 2.4. Identification of TE Differentially Correlated with Responses to Influenza A, HIV, and SARS-CoV-2 Viruses

**Figure 4:**
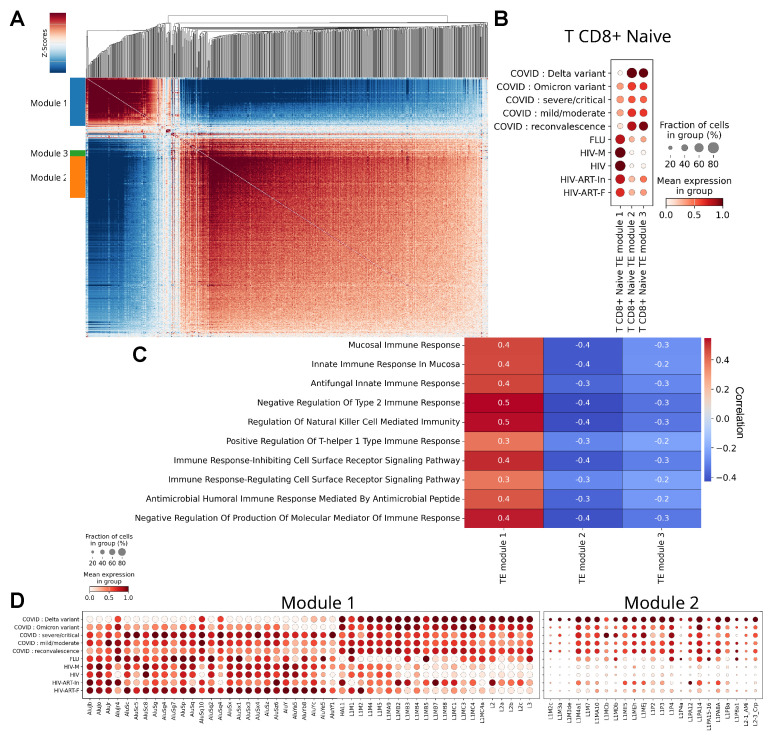
Co-expression modules of TEs in T CD8+ Naive cells are linked to distinct immune pathways and infection outcomes. (**A**) Identification of TE modules and analysis of their expression correlations in T CD8+ Naive cells. (**B**) Expression profiles of the identified TE modules across different cohorts within T CD8+ Naive cells. (**C**) Association between TE module expression and the activity of immunological functional pathways in T CD8+ Naive cells. (**D**) Expression of TE sets characteristic of specific modules across different cohorts within T CD8+ Naive cells.

To better understand the TE expression profile in the most transcriptionally variable cell type (T CD8+ Naive cells) depending on the viral infection variant, an analysis was conducted to elucidate the role of gene subsets in relation to specific functions or processes. Further investigation of transcriptomic differences in immune cells from patients across all studied cohorts was performed using GO analysis and pathway enrichment based on scRNA-seq data (Figure 4A,C). This study analyzed 33,613 genes and 1070 TEs from 451,671 cells, which were divided into three modules (the full gene list is provided in Table S1), with TEs specifically expressed in T CD8+ Naive cells.

As is visible in the dot plot of the averaged relative expression of each TE module across experimental cohorts (Figure 4B), Module 1 exhibits high expression in the “HIV”, “HIV-M”, “HIV-ART-In”, “HIV-ART-F”, and “FLU” cohorts. T CD8+ Naive cells in the “COVID: mild/moderate”, “COVID: severe/critical”, ”COVID: Delta variant”, “COVID: Omicron variant”, and “COVID: reconvalescence” cohorts were characterized by the most significant expression of TEs from Modules 2 and 3. TE expression profiles in patients with influenza A, HIV patients post-ART, and the “COVID: mild/moderate”, “COVID: Delta variant”, and “COVID: Omicron variant” cohorts were enriched, to varying degrees, with TEs from both modules. Naive T-lymphocytes from the “HIV” and “HIV-M” cohorts were distinguished by the expression of only Module 1.

The biological interpretation of cell affiliation with a particular module was based on identifying the expression of genes and their cascades associated with specific processes. It was previously shown that virus-induced TE expression across the genome often occurs near antiviral response genes [31,37]. Therefore, we analyzed the association of TEs with specific molecular processes, considering the proximity of the genes, using the generated combined matrix for determining both TE activity and the cellular transcriptomic profile (Figure 1A). A Gene Ontology analysis was performed for genes expressed in T-cells, grouping them into functional pathways. Functional enrichment analysis revealed the main biological processes involving TEs from Module 1. These included processes of mucosal immune response formation; innate mucosal immune responses; antifungal innate immune responses; negative regulation of type 2 immune responses; NK cell-mediated immune responses; positive regulation of T-helper type 1 cells; signaling pathways of receptors regulating and inhibiting various cellular processes; antimicrobial peptide-mediated antimicrobial immune response; and negative regulation of immune response mediator production (Figure 4C). This indicates that the genes of this module belong to processes of a generalized immune response. Interestingly, Modules 2 and 3 were characterized by a significant decrease in the expression of genes involved in all the aforementioned molecular processes. Modules 2 and 3 are correlated with each other, although the composition of Module 3 is very small.

Despite the general correlation of TE module expression with cell type, variations in this correlation are observed between different functional pathways. A correlation was identified between TE expression and functional pathways combining genes involved in T-cell immune function (Figure 4C). T CD8+ Naive cells from patient groups with influenza A and HIV were characterized by the most significant expression of TEs from Module 1, enriched with markers for the formation of both non-specific and adaptive pro-inflammatory antimicrobial and antifungal humoral and cellular immune responses (Figure 4C), indirectly suggesting enhanced T-cell cytotoxicity in these infections. In contrast, T CD8+ Naive cells from COVID-19 patients were distinguished by a reduction in immune reactions, as indicated by the more pronounced expression of TE Modules 2 and 3 in these cells (Figure 4B,C). Judging by the enrichment with genes from Modules 1 and 2, the greatest reduction in protective immune functions within the pool of precursor effector cytotoxic T-lymphocytes was observed in samples from patients in the “COVID: reconvalescence” and “COVID: Delta variant” cohorts. In the first case, this likely indicates a decrease in immune system intensity due to reduced overall viral load in recovering patients. In the second case, during infection with the SARS-CoV-2-Delta strain, an immunosuppressive state of T CD8+ Naive cells was observed, limiting effective antiviral protection. The reasons for this phenomenon may lie in the unique influence of this strain on gene expression across different cell subtypes, which could be important from the perspective of studying COVID-19 pathogenesis.

A detailed analysis of the composition of differentially active modules 1 and 2 shows that the first module consists of SINE and LINE elements, while the second is composed of LINE elements (Figure 4D). It was found that the expression of Module 1 is significantly increased, and the expression of Module 2 is decreased in cases of influenza A and HIV infection. Furthermore, sample groups from patients with these viral infections are enriched with SINE elements, specifically the L1-dependent, Pol III-transcribed Alu retrotransposon family. No significant differences in TE expression profiles were found between untreated HIV patients (“HIV”) and those with a high viral load (“HIV-M”), both characterized by enrichment of SINE and, to a lesser extent, LINE elements from Module 1. In HIV-infected patients with partially suppressed infection due to antiretroviral therapy (“HIV-ART-In”), a uniform enhancement of the LINE element expression profile from both Modules 1 and 2 is observed compared to untreated individuals.

Based on the data presented in Figure 4D, it can be concluded that the results of SINE element expression in HIV patients after antiretroviral therapy are counterintuitive: it was expected that their expression in patients from the “HIV-ART-F” cohort, with fully suppressed viral infection, would be at the level of the healthy control; in reality, however, it was higher than in other HIV cohorts. It is important to note here that ART includes inhibitors of reverse transcriptase (RNA-dependent DNA polymerase), protease, and integrase, and does not directly affect the activity of the Tat protein. The increased level of SINE element expression in HIV patients after ART likely indicates an enhanced lytic capacity of the pro-inflammatory CD8+ cell phenotype. This suggests the occurrence of previously described ART-induced accumulation of HIV-specific CD8+ T-cells aimed at destroying infected cells and/or preventing viral spread [38,39,40], indirectly supporting the potential involvement of TEs from Module 1 in the functioning of this specific cell population.

Peripheral blood mononuclear cells from COVID-19 patients are uniformly enriched with TE from Module 1, while the expression of genes from Module 2 is higher than in HIV patients. A different expression pattern was observed in patients infected with the SARS-CoV-2-Delta strain: in samples from the “COVID: Delta variant” cohort, the expression of Module 2, as well as LINE elements from Module 1, was increased, while the expression of SINE elements from this module was decreased. The highest expression of regulatory LINE elements in PBMCs of COVID-19 patients was found in the ”COVID: Delta variant” cohort, and to a lesser extent in the “COVID: Omicron” cohort. This is not observed with other coronavirus strains. It is worth noting that LINE element retrotransposition can be directly suppressed by coronavirus proteins; however, the relevance of this phenomenon to the experimental conditions of this work is unclear, as the number of ACE2 receptor molecules on the surface of PBMCs is significantly lower than on the surface of bronchial and lung epithelium [41].

### 2.5. Identification of the LINE Element and Transcription Factor Co-Expression Network

The differential expression of TEs, dependent on the type and stage of viral infection, may broadly reflect changes in the host cell’s transcriptional machinery aimed at countering the virus. It is known that TEs contain cis-regulatory sequences recognized by host TFs and RNA polymerases, which are necessary for hijacking the host’s transcriptional resources for their own replication [42]. This explains the utilization of TE regulatory sequences to modulate the host genome’s transcriptional networks. TEs are known to influence gene transcription in several ways [43]: firstly, by introducing new enhancers or promoters for cellular genes; secondly, by modulating the 3D chromatin structure; and thirdly, by creating new nuclear long non-coding RNAs (lncRNAs), generating new transcription factors through the fusion of DNA-binding domains from their transposase, and by silencing neighboring genes. Furthermore, many TE groups contain multiple TF binding sites [44,45].

**Figure 5:**
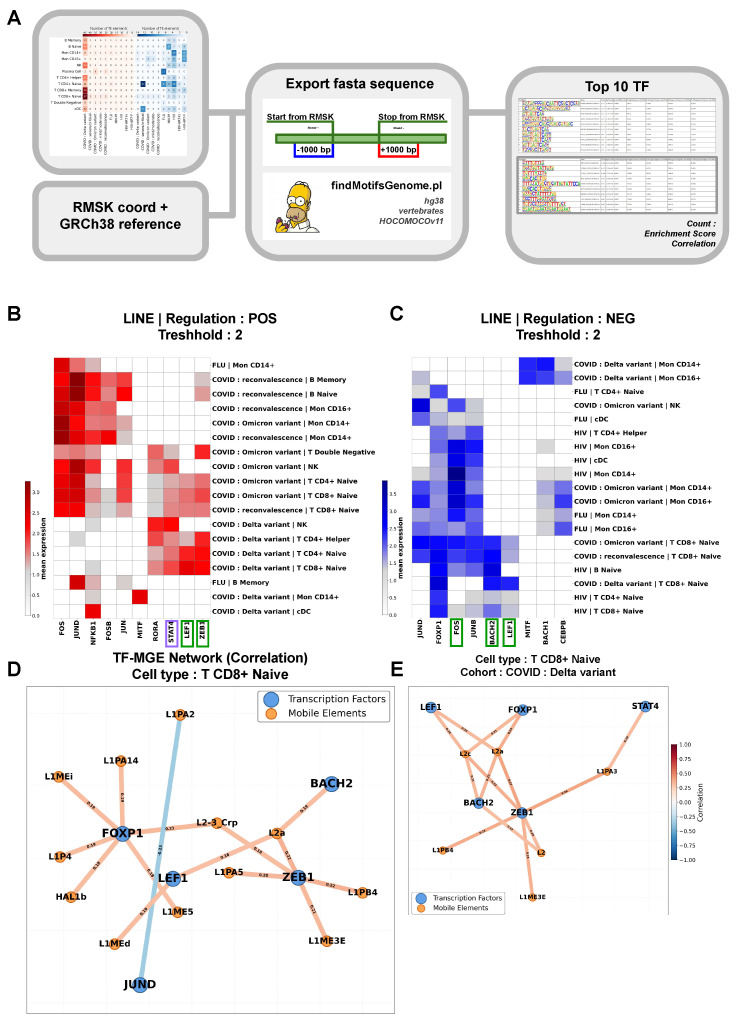
LINE and TF co-expression networks in viral infections. (**A**) Study design includes: obtaining the list of LINE TEs from Figure 3C data; using RMSK data and the GRCh38 reference genome to obtain TE coordinates and sequences; extracting promoter regions from the reference genome; running Homer (findMotifsGenome.pl); and obtaining a list of motifs (top 10) for each unique cohort and cell type combination. (**B**,**C**) Heatmaps displaying high and low enrichment of TF gene-LINE element interaction motifs in PBMCs from patients with viral infections. (**D**) Reconstructed co-expression networks of LINE elements and TOP-5 differentially expressed TFs, identified from the analysis of heatmaps B and C, in T CD8+ Naive cells. (**E**) Co-expression networks of LINE elements and TOP-5 differentially expressed TFs, identified for T CD8+ Naive cells of the “COVID: Delta variant” group. Orange lines indicate positive expression correlation; blue lines indicate negative correlation. Green borders highlight TFs characteristic of both highly and lowly regulated LINE elements. The purple border marks a TF characteristic; in this cell type, these are exclusively shown for the “COVID: Delta variant” cohort.

This section of the work presents a detailed investigation of the features of the co-expression enrichment of TF binding motifs and the most active LINE family retrotransposons during viral infections. Using RMSK data and the reference genome, a list of TF-LINE interaction motifs was extracted (Figure 5A). Based on these data, an expression matrix was created, and its analysis yielded a TOP-10 list of motifs for each unique cohort–cell type combination. As shown in Figure 5B, PBMCs from patients with diseases caused by influenza A virus and SARS-CoV-2 (Omicron and Delta variants) are the most enriched with LINE-TF binding motifs. Most peripheral blood immune cells from patients with COVID-19 (Omicron variant) or convalescents are enriched with LINE binding motifs for TFs involved in generalized Th1 immune responses to inflammation and stress (FOS, JUND, NFKB1, FOSB, JUN). Alongside motifs enriched for non-specific Th1 inflammatory response TFs, T-cells (T-double-negative, T CD4+ Naive, T CD8+ Naive) and NK cells are enriched with motifs for LINE family members and TFs involved in IFNγ-mediated Th1 responses (STAT4) [46] and the formation of Th2 and Th17 responses, such as RORA [47], and ZEB1, also implicated in the formation of a pro-inflammatory autoimmune phenotype [47,48]. Additionally, naive T-lymphocyte fractions are enriched with motifs responsible for binding to TF genes that are markers of their naive state and responsible for their differentiation potential and proliferation - ZEB1 [49,50] and LEF1 [51,52].

Interestingly, the monocytic and B-cell compartments of peripheral blood from patients with influenza A, COVID-19, or convalescents are enriched only with motifs specific to FOS, JUND, NFKB1, FOSB, JUN. A different pattern is observed in T CD4+ Helper, T CD4+ Naive, T CD8+ Naive, and NK cells from patients who had the Delta variant of COVID-19. Although PBMCs from patients with SARS-CoV-2-Delta are more enriched with LINE elements than other cohorts (Figure 3C), we did not find enrichment of LINE-TF binding motifs for TFs regulating non-specific inflammation (FOS, JUND, NFKB1, FOSB, JUN) in the T-lymphocytes of this cohort. However, these cell groups showed enrichment for LINE binding motifs with TFs involved in pathogenic Th1 and Th17 differentiation (RORA, STAT4, ZEB1). Furthermore, CD14+ monocytes from the “COVID Delta variant” cohort were enriched with LINE binding motifs for the MITF gene, a proposed potential driver of transcriptomic changes in COVID-19 pathogenesis and a marker of COVID-19 severity [53].

A reduction in the content of common LINE-TF binding motifs compared to their level in the healthy participant group was detected in immune cells across all studied cohorts (Figure 5C). Interestingly, CD14 and CD16 monocytes from the “COVID: Delta variant” cohort were characterized by a reduced number of binding motifs for LINE family TEs and the genes MITF (a transcriptional activator), BACH1 (which competes for binding in the promoters of many antioxidant genes; [54]), and the pioneer TF CEBPB, capable of “opening” closed chromatin regions, thereby enabling the binding of secondary factors and initiating regulatory pathways [55]. Additionally, T CD8+ Naive cells from this cohort showed a reduction in the content of LINE binding motifs for the genes FOXP1, BACH2, and LEF1, which are involved in T-cell differentiation [56,57]. As seen in Figure 5C, naive T-cells from different cohorts exhibit specific signatures of LINE-TF motif enrichment. For instance, cells from the “COVID: Omicron variant” and “COVID: reconvalescence” groups show a reduction in the content of motifs for both FOXP1, BACH2, LEF1, and genes involved in AP-1 immune activation (FOS, JUND, JUNB). For patients from HIV-related cohorts, no reduction in JUND binding motifs was found, and influenza A is not associated with a decrease in the level of LINE motifs with BACH2 and LEF1.

When assessing virus-specific immune responses based on the expression enrichment of LINE family TE-TF binding motifs, diversely directed involvement was discovered, for example, for the MITF gene in CD14 monocytes of the “COVID: Delta variant” cohort, indicating the presence of both highly and lowly motif-enriched TF gene sequences for different LINE family members. Therefore, an assessment of the correlation between the expression of the TOP-5 TFs, for which LINE binding motifs are most differentially expressed in various viral infections, and individual LINE family members was performed (Figure 5B,C). The analysis was conducted for T CD8+ Naive cells, which play an important role in antiviral immunity, exhibit the most differential levels of TE expression (Figure 3C), and have the most differential levels of LINE-TF motif enrichment depending on the type of viral infection (Figure 5B,C). The greatest co-expression variability with LINE activity was identified for TFs involved in maintaining the naive phenotype (*ZEB1, LEF1, BACH2*) and sustaining immune activation involving AP1 (*FOXP1 and JUND*). This corresponds to the phenotype and functions of T CD8+ Naive cells. Interestingly, most TEs involved in co-expression networks with these TFs belong to the L1 or L2 families. Only for one TF in the TOP-5-the pleiotropic factor *JUND*-was a negative co-expression correlation with one LINE family member, L1PA2, shown. It is known that the activity level (methylation) of the L1PA2 is inversely proportional to the induced level of chromosomal aberrations in cells [58]. It is likely that this TE mediates genomic instability under unfavorable conditions, and JUND is involved in implementing protective regulatory cellular responses of T CD8+ Naive cells to genotoxic stress. JUND showed the greatest variability in the content of binding motifs with TEs among the studied cohorts and cell types, indicating varying degrees of involvement of LINE member-mediated transcriptional regulation of this TF in PBMCs depending on the pathogen type and severity of the viral load. This may be of interest for TE activity-based approaches to diagnosing viral infections, assessing therapy quality, and predicting disease outcomes. The other TFs highly involved in the co-expression networks-*ZEB1, LEF1, BACH2*, and *FOXP1*-are characterized by positive correlation links with LINE family representatives. The greatest number of retrotransposon co-expression links was found for FOXP1-the expression of this TF gene, which supports the naive state of T-cells [59], positively correlates with the expression of L1ME1, L1PA14, L2-3_Crp, L1P4, HAL1b, and L1ME5, most of which we previously assigned to Module 2 (Figure 4D), highly expressed in T CD8+ Naive cells of SARS-CoV-2 cohorts (Figure 4B) and having low association with immune defense activation. Interestingly, for L2a, which we previously assigned to Module 1, enriched with immune defense signaling pathways (Figure 4D), positive co-expression links were identified with three TFs simultaneously-*ZEB1*, *LEF1*, and *BACH2*. The expression of L2-3_Crp positively correlates with the expression of *ZEB1* (which shows high motif enrichment in T CD8+ Naive cells) and FOXP1 (which shows a low degree of interaction with TEs in almost all cell types during viral infections; Figure 5C). This may indicate the involvement of these TEs in maintaining the naive status of CD8+ T-lymphocytes. It can be assumed that these specific TFs regulate the biological functions, including antiviral immune activity, of TEs in T CD8+ Naive cells. The presence of a large number of overlapping co-expression links suggests high variability in the retrotransposon transcriptional signatures of CD8+ T-lymphocytes during the formation of antiviral responses.

It is known that severe SARS-CoV-2 infection is associated with increased signs of CD8+ T-cell exhaustion [60,61]. Analysis of the LINE element and TF co-expression profile in our single-cell RNA sequencing dataset revealed that the T CD8+ Naive cell population exhibited the greatest heterogeneity, with the highest degree of differences observed in the “COVID: Delta variant” group (Figure 5E). This T CD8+ Naive cell phenotype was characterized by a high level of DE LINE-TF common binding motifs for *ZEB1, LEF1, BACH2, FOXP1*, as well as *STAT4*, which plays a key role in the JAK-STAT-mediated development of immune responses by enhancing Th1 cell differentiation, cytotoxicity, and IFN-gamma production by immune cells [46,62]. For this cohort, positive co-expression links were identified between *BACH2, LEF1*, and *FOXP1* and representatives of the LINE2 family (L2, L2a, L2c). In addition to L2 representatives, positive correlation links were identified for ZEB1 with L1ME3E, L1PA3, L1PB4, and for STAT4 with L1PA3.

## 3. Discussion

This study conducted a cohort analysis of TE expression in the single-cell RNA sequencing data of peripheral blood mononuclear cells from patients with viral infections (influenza A, HIV, COVID-19), aiming to identify potential biomarkers for diagnosing and predicting the course of viral diseases, as well as for assessing the effectiveness of antiviral therapy. Unique TE expression signatures in PBMCs were identified, enabling the differentiation of disease severity and prediction of the course of SARS-CoV-2 infection (increased expression of L1MDA, L1MA2, L1PA16) and the identification of the SARS-CoV-2-Delta strain (increased expression of L1MDA, L1MA2, L1PA16, Charlie21a; decreased expression of Alu family and DNA transposons). Furthermore, we were able to distinguish the completion status of antiretroviral therapy in HIV patients. A specific expression pattern in the PBMCs of patients who did not complete ART was the high differential expression of L1MDA, L1MA2, L1PA16, and Charlie1a, which was not observed in other HIV patient cohorts.

The study identified human blood cell types most susceptible to changes in TE expression during viral infections, as well as differentially expressed TE signatures characteristic of different PBMC cell types in viral diseases of varying origin, duration, and severity. The distribution and expression level of LINE elements in blood cells were found to be more dependent on the specific characteristics and nature of the viral disease compared to the expression of SINE elements. The most variable TE expression profile, dependent on the pathogen type and disease severity, was found in the precursors of cytotoxic T-lymphocytes responsible for the antiviral response–T CD8+ Naive cells.

Additionally, we analyzed the activity of LINE retrotransposons in these cells during different viral infections based on the enrichment of their binding motifs with transcription factors. It is known that TFs can bind to regions of active retrotransposons to regulate the expression of genes necessary for retrotransposition (e.g., reverse transcriptase) or, conversely, to suppress this activity. In turn, accessible LINE sequences can serve as a source of enhancer, silencer, or promoter sequences for TFs, influencing their activity [15,19]. We found that in T CD8+ Naive cells, the TFs most highly involved in co-expression networks with LINE elements were *ZEB1, LEF1, BACH2, FOXP1*, and *JUND*. In T CD8+ Naive cells of the “COVID: Delta variant” group, specific features of LINE transcriptional regulation were identified, involving the high involvement of the STAT4 gene (regulating interferon-gamma synthesis) and LINE2 family members in the TF-LINE co-expression networks. Finally, we identified JUND as the TF interacting most variably with LINE elements across different PBMC cell types, depending on the specific characteristics and nature of the viral disease. This suggests JUND as a potential diagnostic biomarker, including for assessing therapy quality and predicting disease outcomes, or as a therapeutic target for viral infections accompanied by genomic instability and defects in antiviral responses.

The involvement of various TEs in the antiviral response is now considered proven, due to their participation in a wide spectrum of physiological and pathological immune reactions. Firstly, increased transposon expression in host cells occurs near antiviral response genes and is present in all studies, regardless of the virus, species, or host cell tissue type [37]. Secondly, transcripts derived from retrotransposons exert profound and multifaceted effects on immune system function [63], including the activation of “viral mimicry” mechanisms. Virus-like repetitive TE sequences act similarly to viral pathogen-associated molecular patterns (PAMPs) to stimulate antiviral defense [32]. Depending on the transcript type, LINE1 can activate signaling pathways leading to stimulation of type I IFN responses involving RIG-I and MDA5 [64], and cGAS [65]. Furthermore, L1 elements can act as cis-regulators, serving as enhancers that activate IFN-stimulated genes and modulate its signaling pathway [65,66]. Interestingly, active retrotransposons can be directly responsible for a generalized antiviral response. For instance, the mouse LINE1 Lx9c11, which regulates the Schlafen gene family via the non-coding RNA Lx9c11-RegoS, was shown to control the antiviral immune response and promote mouse survival during viral infection [67].

Alongside transient transcriptomic responses, viral infections, including SARS-CoV2, influenza A, and HIV, induce epigenetic remodeling of the host genome as part of the immune response. DNA methylation and histone modifications induced by viral infection persist even after the disease ends and the virus is completely eliminated [68,69]. Recent studies have shown that virus-infected cells often exhibit increased TE expression, which can be triggered by cellular stress from the viral infection, as well as by enhanced global DNA demethylation [70,71]. The mechanism of HIV’s influence on TEs’ activity has long been known-the activation of TE transcription by the viral Tat protein was demonstrated as early as 1992, with the Tat protein enhancing the activity of a cellular transcription factor from the major TF group–TFIIIC [72]. HIV infection can be a stimulus for transitioning TEs into a transcriptionally active state, thereby leading to antigen presentation on the surface of infected cells and a subsequent immune response [28]. LINE-1 can increase endogenous interferon levels, leading to the expression of regulatory RNAs and subsequent suppression of HIV. Interestingly, LINE-1 ORF1p can be incorporated into HIV virions, but its biological significance is currently unknown. Therefore, it is unclear whether LINE-1 can directly promote or suppress HIV infectivity [73]. HIV can also directly impact LINE-1 retrotransposition, but evidence on this is quite contradictory. For instance, some suggest that viral components can directly enhance LINE-1 activity and influence its activity by neutralizing ISG proteins and via regulation of the host innate immune system [74]. ART is the primary method for controlling and suppressing HIV-1 to undetectable levels to prevent AIDS [75]. Interestingly, ART may have little effect on pathological methylation patterns corresponding to a transcriptionally active state and, consequently, on TE activity in HIV [76]. Moreover, the increased TE activity in immune cells of HIV “elite controllers”-individuals who maintain undetectable plasma viremia without ART-may be the cause of their resistance to HIV-1 [77].

Analysis of the expression of various immune cells in the PBMS after HIV infection is based on the nature of the antiviral mechanisms involved in this infection. It is known that, upon HIV infection, CD4+ T cells are also activated, for example, by mechanisms such as the activation and cytotoxicity of CD8+ T cells. Together, these mechanisms illustrate the complex interplay of genetic, immunological, and epigenetic contributions to viral control.

During influenza A virus infection, patients also exhibit changes in retrotransposon transcriptional levels; the variability of the response to viral infection and viral load may correlate, among other factors, with the transcriptional activity of TEs and their regulating host cell TFs. For example, 204 TEs with increased expression and 7 elements with decreased expression were discovered during influenza infection; the interferon response system and the KRAB-ZNF signaling pathway are believed to be involved [31]. It is worth noting that non-coding RNA from Alu elements primarily acts as a repressor for viral RNA polymerase II promoters: it forms a triple complex with RNA polymerase II and its recognizable promoters, binding strongly and hindering transcription initiation [78]. The influenza virus may limit interactions with TEs induced during infection, likely by isolating most of them in or around the nucleus, thus affecting their subcellular distribution and immunostimulatory potential [32].

SARS-CoV-2 infection causes long-term (for several months after recovery) changes in methylation patterns [29], where increased retrotransposon expression is a protective reaction of the body. To avoid excessive immune system activation, SARS-CoV-2 employs various protective mechanisms, such as encoding different proteins to control L1 mobility, inhibiting TE transcription activation through interaction with various chromatin-binding factors, which limits the type I IFN response and antiviral immunity [27,30]. The involvement of SINE elements (Alu) in regulating the response to SARS-CoV-2 infection is mediated by their presence in the intronic regions of the gene for this virus’s main receptor–angiotensin-converting enzyme 2 (ACE2)-as well as genes involved in the immune response and coagulation system [79]. Depending on the position of Alu elements in the intronic regions of the receptor, the nature of the disease course and the expression level of differentially transcribed genes can vary. Interestingly, in the 5’-region upstream of the ACE2-coding region, an Alu element separates two promoters; furthermore, this gene has a so-called internal promoter from which a transcript encoding its short isoform, insensitive to the SARS-CoV-2 protein, is read [80]. LINE elements in COVID-19 are less studied. Two widely discussed studies exist: one showed that in coronavirus-infected HEK293 cells with a plasmid containing a LINE1 element, LINE1-dependent integration of DNA copies of coronavirus RNA into the genome occurred at LINE endonuclease recognition sites (in 29% of cases, integration occurred into gene exons) [81], while the other found retrotransposition only during cell infection with SARS-CoV-2 virus, but not during transfection with its mRNA [82]. However, another study, conversely, found no single case of integration of DNA copies of viral RNA fragments into the cell genome during SARS-CoV-2 infection of HEK293T cell lines using nanopore sequencing data analysis, despite noting dozens of L1 element integration sites in the cells [83]. Thus, the question of the likelihood of SARS-CoV-2 gene integration into the host genome with the participation of LINE elements remains open.

## 4. Materials and Methods

In this work, we analyzed and integrated data from several sources, including studies of the immune response in various pathologies such as COVID-19, HIV, and influenza A. The analysis utilized data obtained by scRNA-seq.

### 4.1. Sample Collection Dates and Patient Groups

This study was conducted in accordance with the principles of the World Medical Association’s Declaration of Helsinki. Participants were enrolled after they or their legal representatives provided written informed consent for participation in the research, the collection of biological samples, and the processing of personal data. Furthermore, the studies were approved by the ethical committees of the respective institutions.

We utilized both in-house and public scRNA-seq data of peripheral blood mononuclear cells from patients with various infections. The in-house data include sequencing data from blood samples of patients diagnosed with COVID-19 and control samples from healthy individuals. Publicly available sequencing data from patients diagnosed with influenza A, COVID-19, and HIV were downloaded. All publicly available datasets included control samples from healthy individuals.

The following publicly available data from HIV patients were selected: The first sample set was collected as part of the Yale University HIV Associated Reservoirs and Comorbidities (HARC) project between January 2018 and March 2020. The second HIV sample set (from both healthy donors and donors living with HIV) was collected at the UCSD AntiViral Research Center from 2018 to 2019. The third HIV sample set was obtained from existing cohorts of donors with HIV-1 and seronegative individuals stored at the Duke Human Vaccine Institute; data collection was conducted over several years, up to 2022. An additional set of samples from healthy donors was obtained from a study on Sjögren’s syndrome conducted between 2019 and 2020 [84]. Blood cell expression data from COVID-19 patients were supplemented with results sequenced at Asan Medical Center, Severance Hospital, and Chungbuk National University Hospital during the pandemic, while data for influenza A patients were collected earlier, from December 2015 to April 2016.

Based on the available sample metadata, the groups were subdivided into cohorts. The study used samples from patients across 11 cohorts (Table 1): conditionally healthy individuals (the “Healthy” cohort, whose participants had laboratory-We confirm. negative tests for the viruses under investigation); 5 cohorts of patients infected with the SARS-CoV-2 virus (the “COVID” groups); 4 groups of patients with HIV (the “HIV” groups); and a group of individuals diagnosed with influenza A virus via a rapid antigen test (the “FLU” group).

The in-house data, comprising samples from SARS-CoV-2 patients obtained at the Federal Clinical Center of FMBA, included a cohort with mild/moderate disease (the “COVID: mild/moderate” cohort, 16 samples) according to the WHO guidelines and deposited in the NCBI BioProject repositories with accession numbers PRJNA1164162 and PRJNA1370311. Furthermore, we investigated 13 blood samples from patients with the SARS-CoV-2-Delta variant, 4 samples for the SARS-CoV-2-Omicron variant, 6 samples from recovering patients (the “COVID: Delta variant”, “COVID: Omicron variant”, and “COVID: reconvalescence” cohorts, respectively), and 9 healthy patients (the “Healthy” cohort).

Participants with SARS-CoV-2 from public sources were divided into cohorts with mild/moderate (the “COVID: mild/moderate” cohort, four samples) and severe/critical (the “COVID: severe/critical” cohort, four samples) disease. Participants in the HIV study were divided into the following cohorts: a cohort of 3 patients not receiving antiretroviral therapy (ART) (the “HIV” cohort), a cohort of 3 patients with a high viral load (the “HIV-M” cohort), a cohort of samples from 2 patients with a partially ART-suppressed infection (the “HIV-ART-In” cohort), and a cohort consisting of 12 patients with a fully ART-suppressed infection (the “HIV-ART-F” cohort). The influenza A-infected cohort (the “FLU” cohort) included samples from five patients. Public data also included data from 17 healthy patients (the “Healthy” cohort).

### 4.2. PBMC Collection

Blood samples were collected during the following periods: public data were collected from January 2018 to March 2020 for patients from the HIV study cohorts and from December 2015 to April 2016 for patients with influenza A. Public samples from COVID-19 patients were collected after the start of the COVID-19 pandemic, using data obtained from the Federal Clinical Center of FMBA from June 2020 to August 2021 (covering the early stages of the COVID-19 pandemic and the spread of the SARS-CoV-2 Delta variant). Sample collection and processing methods varied depending on the data cohort under investigation. Peripheral blood was collected into EDTA tubes and processed within 1–4 h after collection. The blood samples were then subjected to density gradient centrifugation using Ficoll (Invitrogen, Waltham, MA, USA), after which PBMCs were isolated, washed, and used immediately while stored on ice, or frozen and stored until analysis. Prior to analysis, the thawing and removal of dead cells (including the use of magnetic beads) were performed and viability was assessed. After thawing, all samples used for analyses demonstrated high viability, averaging approximately 90%.

### 4.3. Single-Cell RNA Sequencing

Library preparation and sequencing methods varied between the different datasets; however, the 10× Genomics platforms and sequencing on Illumina (San Diego, CA, USA) instruments using the Chromium Single Cell 3’ protocol were used in all cases (Table 1). For single-cell gene expression analysis, the 10× Genomics platform versions v2.0, v2, v3, and v3.1 were used. Between 2000 and 10,000 cells per sample were encapsulated in oil droplets to form GEMs (Gel Bead-in-Emulsions). Reverse transcription of mRNA was performed with the addition of Unique Molecular Identifiers (UMIs) and cell barcodes to cDNA, followed by cDNA amplification and library construction. cDNA fragmentation (200–300 base pairs), adapter ligation, and PCR amplification were performed prior to sequencing. Sequencing was carried out on Illumina (San Diego, CA, USA) HiSeq 4000/HiSeq2500, NextSeq 550/NextSeq2000, and NovaSeq 6000 platforms. The read parameters were as follows: Read 1-26 base pairs; i7 index-8 base pairs; Read 2-98 base pairs. Sequencing depth was approximately 50,000–60,000 reads per cell. Despite variations in experimental approaches, all studies ensured high sequencing depth and robust quality control.

### 4.4. Reference Genomes and Annotations

For single-cell transcriptomic analysis, reference genomic and annotation data were integrated from multiple established sources. The human reference genome (GRCh38, https://cf.10xgenomics.com/supp/cell-exp/refdata-gex-GRCh38-2024-A.tar.gz (accessed on 15 October 2024) ) and a core gene annotation were sourced from the standard 10× Genomics (Pleasanton, CA, USA) Cell Ranger package (refdata-gex-GRCh38-2024-A), which is optimized for the 10× Genomics processing pipeline.

To enable a comprehensive transcriptome analysis, including the detection of transcriptionally active retrotransposons and other TEs, additional annotation resources were incorporated. High-confidence gene annotations were obtained from the GENCODE https://ftp.ebi.ac.uk/pub/databases/gencode/Gencode_human/release_30/gencode.v30.annotation.gtf.gz (accessed on 15 October 2024)), providing an up-to-date and detailed catalog of gene loci. Annotations for genomic repetitive elements, including the classification and genomic coordinates of TEs, were retrieved from the authoritative RepeatMasker catalog for the hg38 assembly via the UCSC Genome (http://hgdownload.soe.ucsc.edu/goldenPath/hg38/database/rmsk.txt.gz (accessed on 15 October 2024)).

Using these integrated annotations (a merge of GENCODE and RepeatMasker), a custom genomic index was built using the dedicated scTE_build tool. This index enables the simultaneous quantification of both protein-coding gene expression and the diverse families of repetitive elements within a unified analysis framework, which is essential for investigating the role of TEs in transcriptional regulation at single-cell resolution.

### 4.5. Primary Data Processing and Analysis

Data processing included demultiplexing using the bcl2fastq utility (v1.8.4, Illumina (San Diego, CA, USA)), alignment to the human reference genome GRCh38 using the Cell Ranger pipeline (10× Genomics, v8.0.1, https://www.10xgenomics.com/support/software/cell-ranger/8.0 (accessed on 15 October 2024)), and the generation of a gene expression matrix. To increase representativeness and statistical power, the in-house data were integrated with public single-cell datasets.

### 4.6. Generation of Gene Matrices, Filtering, and QC

The obtained expression matrices were processed individually in the ScanPy environment (v1.10.4) [85] (https://github.com/scverse/scanpy (accessed on 15 October 2024)). The following quality control (QC) filters were applied: cells were retained only if they contained between 500 and 5500 genes, and the percentage of mitochondrial reads did not exceed 15%. The filtered gene-barcode matrices were normalized using the ‘pp.normalize_total’ function, and the most highly variable genes (*n* = 3000) were identified using the ‘pp.highly_variable_genes’ method. After filtering and quality control, a single-cell RNA sequencing matrix of peripheral blood mononuclear cells (PBMCs) was generated. Principal component analysis (PCA) was then performed, followed by UMAP transformation based on the first 30 principal components. The quality of data integration was verified by visualizing individual samples on UMAP plots.

### 4.7. Data Integration and Cell Typing

Data integration was performed by sequentially merging the single-cell RNA sequencing data from individual patients into a single annotated object. To ensure data consistency and preserve biological heterogeneity, the Harmony utility was used [86] (https://github.com/immunogenomics/harmony (accessed on 15 October 2024)). Integration was carried out using the pp.harmony_integrate function on the PCA matrix with default parameters. The patient identifier was used as the key to account for batch effects. Major cell types were identified and annotated using the publicly available model COVID19_HumanChallenge_Blood.pkl and the CellTypist tool (v1.6.3) [87] (v1.6.3, https://github.com/Teichlab/celltypist (accessed on 15 October 2024)). Cell clustering was performed based on a neighborhood graph using the Leiden algorithm with a resolution parameter of 2.

### 4.8. TE Matrix Generation

To analyze TE activity at the single-cell level, we used an approach based on integrating scRNA-seq data pre-processed with the Cell Ranger pipeline (10× Genomics) and subsequent processing with the scTE utility (v1.0) [88] (https://github.com/JiekaiLab/scTE (accessed on 15 October 2024)). The scTE tool allows for the direct analysis of repetitive element activity from scRNA-seq data. The process involved the following steps: TE annotation preparation, where an annotation of repetitive elements was created based on the RepeatMasker database. Aligned reads were obtained from the output of the count command within the Cell Ranger tool (10× Genomics, v8.0.1). Cell and gene filtering was performed according to standard default values to minimize technical artifacts.

### 4.9. TE Data Object Integration and Cell Annotation

Integration of the TE data object was performed similarly to the gene data object integration using the Harmony utility. Cell annotations were transferred from the gene data object and subsequently used for the interpretation of the TE data.

### 4.10. Identification of Top 10 Differentially Enriched TE-Transcription Factor Binding Motifs for Each Unique Cohort-Cell Type Combination

To identify potential TE regulators, we performed an analysis of transcription factor (TF) motif enrichment in promoter regions, accounting for DNA strand orientation and spanning 1000 bp from the TE start coordinate. Sequences were extracted from the human GRCh38 genome, and the analysis was performed using HOMER2 v. 5.1 (http://homer.ucsd.edu/homer2/ (accessed on 15 October 2024)) function findMotifsGenome.pl. For each “cohort-cell type-regulation direction” combination, the top 10 significantly enriched motifs with a log2 FC ≥ 0.5 were selected. Based on the obtained data, a matrix of averaged expression for the selected TFs was constructed.

### 4.11. Construction of Co-Expression Networks Between LINE Family TEs and Transcription Factors in T CD8+ Naive Cells

To analyze functional interactions between TFs and LINE family TEs, a co-expression network was constructed based on the data. The analysis was performed for the population of peripheral blood T CD8+ Naive cells. The initial data comprised a MuData multimodal object containing gene and TE expression matrices. From a pre-selected list of TFs associated with LINE promoter regulation and the full set of LINE elements, pairwise Pearson correlations between TF and TE expression levels were calculated. The statistical significance of correlations was assessed using the Benjamini–Hochberg correction for multiple testing (FDR < 0.05). Only statistically significant connections with an absolute correlation coefficient value > ±0.18 were used for network construction. The network structure was visualized using the NetworkX library (https://github.com/networkx/networkx (accessed on 15 October 2024)), where nodes represented TFs (blue) and LINE elements (orange), and edges represented significant correlation links. The edge color reflects the direction and strength of the correlation (red for positive, blue for negative) using a non-linear transformation to enhance contrast.

## 5. Conclusions

The results obtained in this work undoubtedly prove the importance of TE involvement in regulating antiviral immune responses, dependent on the virus type, disease severity, and therapeutic treatment. We successfully identified cell-specific patterns of TE expression and assessed their activity depending on the nature of the infection. Nevertheless, further research is needed to understand the mechanistic nature of TE-mediated antiviral protection in viral diseases of various origins. Firstly, future studies should enhance the sample size both by increasing the total number of participants, which is a limitation of the current study, and by expanding the catalog of viral diseases. Secondly, future research should be conducted considering the immunotyping of patients across all cohorts [89] and standardization of experimental protocols. Thirdly, integration with genetic analyses such as eQTL (expression quantitative trait loci), sQTL (splicing quantitative trait loci), and GWAS, as well as an assessment of the proteome and metabolome, will help predict disease outcomes and evaluate the prospects for the clinical use of these cis-regulators as therapeutic targets in viral infections. Finally, the use of new computational approaches in studies of retrotransposon expression profiles in immune cells will help generate new data on the value of using TEs as biomarkers for diagnosing and monitoring viral infections, as well as for expanding the understanding of the biological role of TEs in the body’s antiviral defense.

## Figures and Tables

**Table 1:** Quantitative representation of the cohorts.

Pathogenic Virus	Cohort	Number of Samples	Data Source	Sequencing Platform
SARS-CoV-2	COVID: mild/ moderate	20 (4 + 16)	Public (PRJNA629752) + in-house (PRJNA1164162, PRJNA1370311)	Nextseq550, NovaSeq6000, NextSeq2000
SARS-CoV-2	COVID: severe/critical	4	Public (PRJNA629752)	Nextseq550, NovaSeq6000
SARS-CoV-2	COVID: Delta variant	13	In-house (PRJNA1164162, PRJNA1370311)	NovaSeq6000, NextSeq2000
SARS-CoV-2	COVID: Omicron variant	4	In-house (PRJNA1164162, PRJNA1370311)	NovaSeq6000, NextSeq2000
SARS-CoV-2	COVID: reconvalescence	6	In-house (PRJNA1164162, PRJNA1370311)	NovaSeq6000, NextSeq2000
-	Healthy	26 (9 + 17)	Public (PRJNA835867, PRJNA662927, PRJNA681021, PRJNA660749, PRJNA629752) + in-house	HiSeq4000, HiSeq2500, NextSeq500, NovaSeq6000, NextSeq2000
Influenza A	FLU	5	Public (PRJNA629752)	NextSeq500
HIV	HIV	3	Public (PRJNA681021)	HiSeq2500, NextSeq500
HIV	HIV-M	3	Public (PRJNA681021)	HiSeq2500, NextSeq500
HIV	HIV-ART-F	12	Public (PRJNA835867, PRJNA662927, PRJNA681021)	HiSeq4000, HiSeq2500, NextSeq500
HIV	HIV-ART-In	2	Public (PRJNA662927)	HiSeq4000, HiSeq2500, NextSeq500

## Data Availability

Publicly available datasets were analyzed in this study. These data can be found in the NCBI BioProject database under accession numbers: PRJNA629752, PRJNA835867, PRJNA662927, PRJNA681021, and PRJNA660749. The newly generated single-cell RNA-seq dataset supporting the findings of this study has been deposited in the same repository under accession number PRJNA1370311. Additional in-house sequencing data (previously generated) were used under accession PRJNA1164162.

## Supplementary Materials

The following supporting information can be downloaded at: https://www.mdpi.com/article/10.3390/ijms27031286/s1.
