# Supplementary material for: Single-Cell RNA-Seq Profiling of Transposable Element Expression in Human Peripheral Blood Cells During Viral Infections
Source: Int J Mol Sci. 2026 Jan 28;27(3):1286. doi: 10.3390/ijms27031286 (PMC12898442; doi:10.3390/ijms27031286)
Supplement: Supplementary file 1 [file ijms-27-01286-s001.zip › ijms-4093079-supplementary.pdf]

International Journal of
Molecular Sciences

MDPI

Article

Single-Cell RNA-Seq Profiling of Transposable Element Expression in Human Peripheral Blood Cells During Viral Infections

O. D. Fateev [1], V. E. Akimov [1], O. V. Glushkova [1], A. V. Bolbat [1], A. V. Abdullatypov [1], O. A. Antonova [1], V. V. Shiryagin [1], N. A. Bugaev-Makarovsky [1], V. S. Yudin [1], A. A. Keskinov [1], S. M. Yudin [1], D. V. Svetlichny [1,*] and V. I. Skvortsova [2]

[1] Federal State Budgetary Institution "Center for Strategic Planning and Management of Biomedical Health Risks" of the Federal Medical-Biological Agency (FSBI "CSP" of FMBA of Russia). 119121, Russia, Moscow
[2] Federal Medical-Biological Agency (FMBA of Russia). 123182, Russia, Moscow
* Correspondence: DSvetlichnyj@cspfmba.ru

1. Supplementary materials

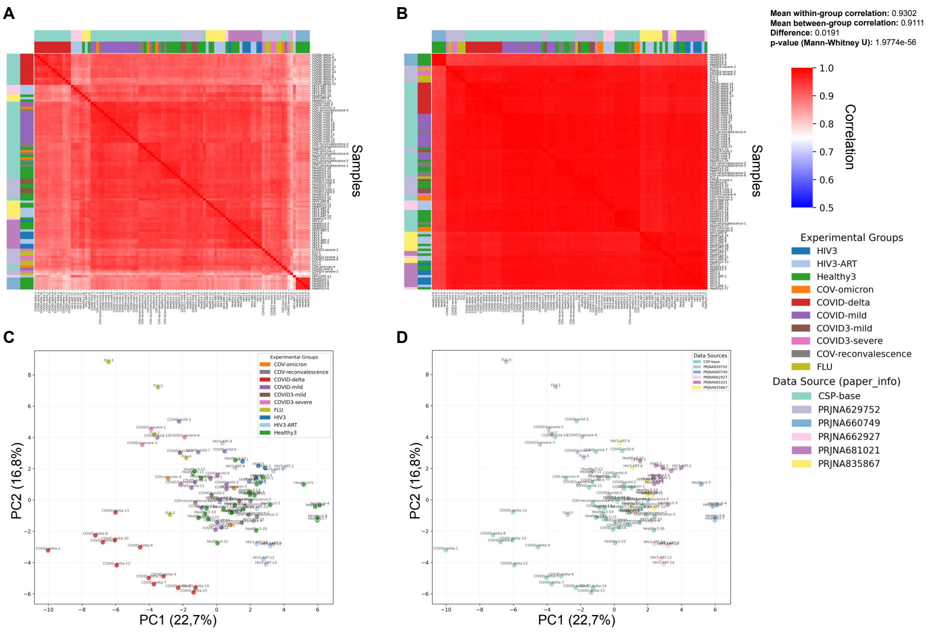

Figure S1. Assessment of batch effects and sample concordance across integrated scRNA-seq datasets. (A) Pearson correlation matrix based on the top 3,000 highly variable genes across all samples. (B) Pearson correlation matrix computed from the TE expression matrix (TE × cell). (C, D) Principal component analysis (PCA) based on the same set of 3,000 highly variable genes. Samples are colored by experimental group in (C) and by dataset of origin in (D). The mean within-group correlation (0.9302) is significantly higher than the mean between-group correlation (0.9111; difference = 0.0191, Mann–Whitney U test, $p = 1.98 \times 10^{-56}$), indicating robust integration with minimal batch effects and strong biological consistency across datasets.

Check for updates

Academic Editor: Firstname Lastname

Received: 25 December 2025
Revised: 20 January 2026
Accepted:
Published:

Copyright: © 2026 by the authors. Licensee MDPI, Basel, Switzerland. This article is an open access article distributed under the terms and conditions of the Creative Commons Attribution (CC BY) license.

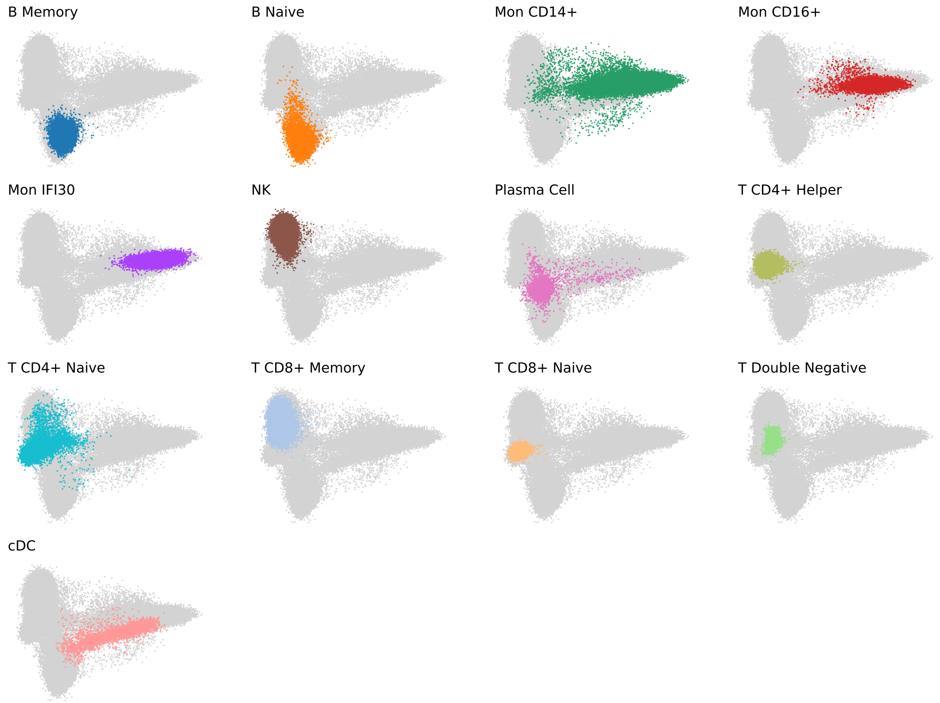

Figure S2. Principal Component Analysis (PCA) plot of the scRNA-seq data of the data used to validate the annotations of cells

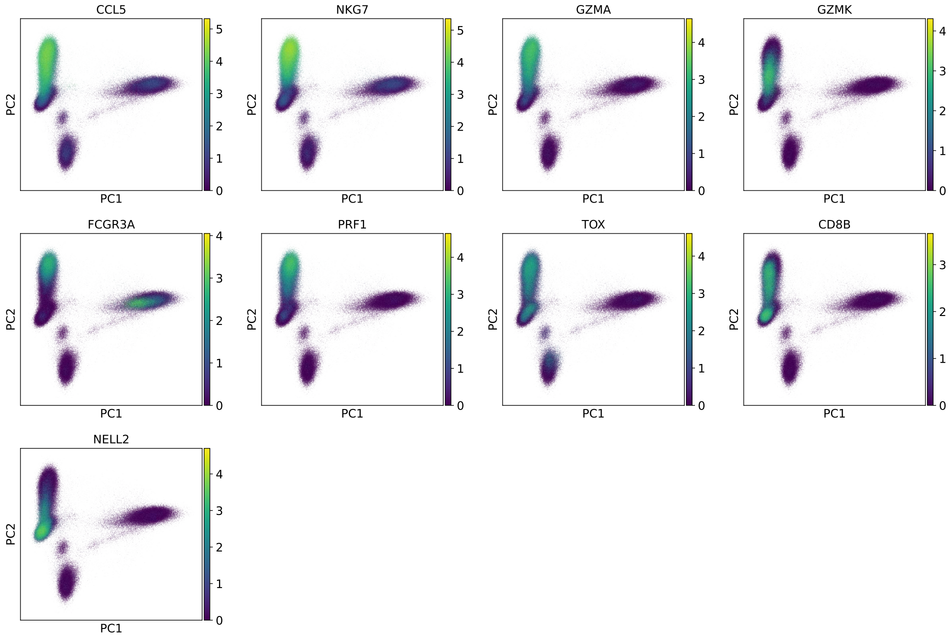

Figure S3. Principal Component Analysis (PCA) plot of the scRNA-seq data with overlaid expression of established T cell subset marker genes, unequivocally defining the annotated populations.

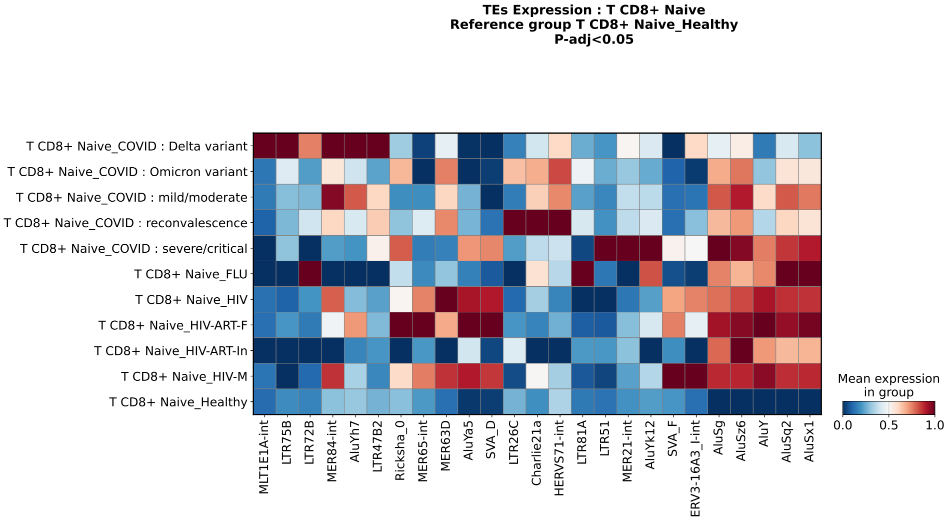

Figure S4. Average normalized expression of the top 3 differentially expressed TE in T CD8+ Naive cells within each cohort. Demonstration of a TE signature specific to each cohort.

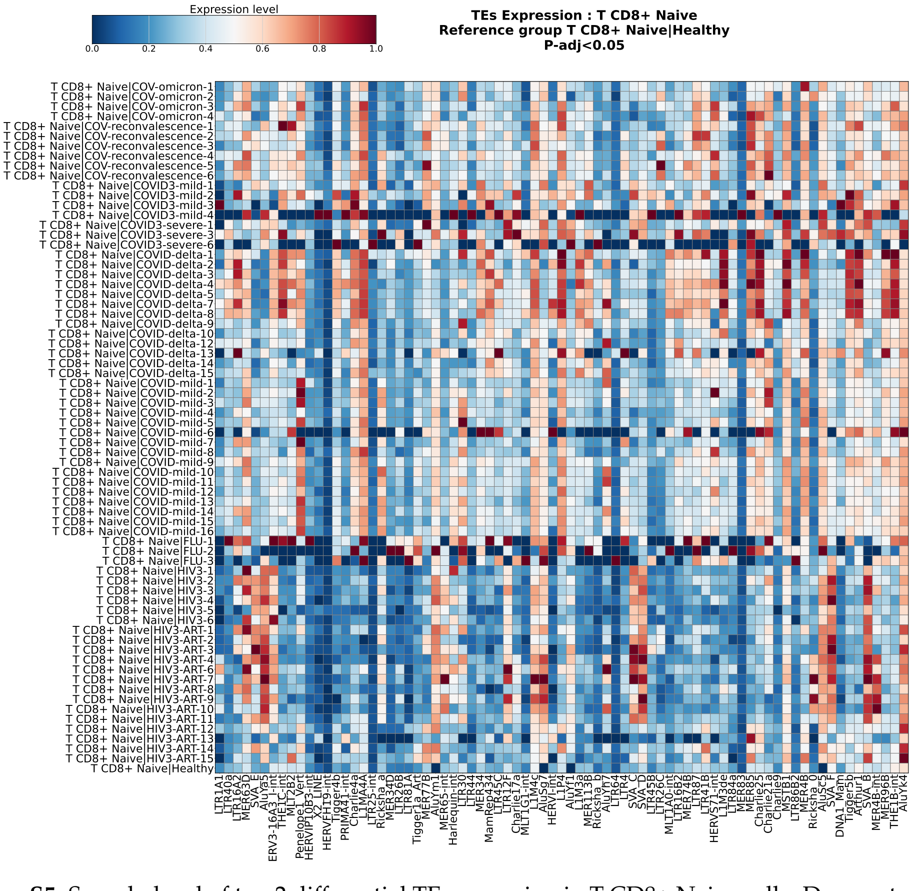

Figure S5. Sample-level of top 2 differential TE expression in T CD8+ Naive cells. Demonstration of a consistent TE activation signature across individual patients for each infection type.

Table S1. Composition of the discovered modules of TEs

Module number	Transposable Elements
Module 1	MER58C, Ricksha_0, SVA_D, MER5A1, MER58B, MER30, AluY, AluSz, AluJb, MLT1D, AluSx1, AluSx, AluSq2, AluSp, SVA_F, AluJo, MIRb, AluSx3, AluJr, AluSz6, L1ME3A, AluSc, AluSc8, AluSg, AluSq, MIR3, AluYa5, L2a, AluSx4, AluSg4, FLAM_C, MIR, L1MC5, L1MB8, FLAM_A, L1ME4a, AluYm1, L1MC4, AluSc5, L2c, L2b, MIRc, AluJr4, AluSg7, AluYb8, L1M5, L1ME1, L1MB7, MER65-int, L2, L1MEd, L1M1, MER41D, L1M2, FRAM, Tigger1, AluYc, L1MB3, HAL1, L1ME4b, L1MC5a, L1PA2, L3, L1M4, L1PA3, L1MC3, AluYj4, L1MEc, Charlie1a, AluSq10, AluYh3, L1MEg, LTR12E, L1ME3Cz, LTR5B, MER2, L1MB4, L1ME2, MER58A, L1MB5, L1MC1, L1PA4, AluSq4, L1PB1, AluYf1, LTR46-int, L1MD1, SVA_B, FAM, AluYk3, LTR, MER3, AluYg6, MER5B, MER20, L1ME3, L1MB2, AluYe5, Tigger3b, L1MD2, MER33, L1ME4c, AluYk4, L1MA9, L1ME3G, L1MEf, MER5A, L1PA5, MSR1, L1MC4a, L1PA7
Module 2	MSTA-int, MLT1O, LTR40a, MLT1A0-int, LTR10F, MLT1D-int, L2-3_Crp, L1PA12, Arthur1, LTR33, L1P2, HERVH-int, LTR7, MER101-int, LTR7B, Tigger5b, LTR82A, Tigger12, L1M7, Charlie17, CR1-3_Croc, Charlie15a, L1P4a, MLT1J1, MLT1E2, L1PA14, LTR16A, Charlie25, L1PBa, MER34A1, Tigger12c, Tigger12A, L1PA8A, MSTB2, L1M2c, HERVK11-int, L1P4, AmnSINE1, Tigger3, L1P3, Tigger5, MER53, Charlie19a, L1MEj, HERVFH21-int, MER57-int, MLT2A1, HERVL-int, MER77, L1MCb, MLT2A2, L2-1_AMi, HERVH48-int, BLACKJACK, LTR33A, Plat_L3, CR1_Mam, HUERS-P3b-int, MER11C, MER94, Cheshire, L1M3a, LTR24C, MER6B, MER41-int, Tigger20a, MER110-int, MER47B, MER31B, MLT1M, Tigger6a, MER52-int, MER63A, L1PA15-16, MER74B, MER44A, MER63B, Tigger10, L1ME5, L1MDb, MER52A, MER51-int, MER4A1, Kanga11a, L1PBa1, L1MEh, L1M3de, MER1A, MER47A, LTR10C, Arthur1B, L1M4a1, Looper, L1MA10, MLT1E1A-int, THE1A-int
Module 3	MLT2D, LTR78, THE1A, OldhAT1, MER68, MLT2B4, ERVL-B4-int, MLT1G1, MLT1J2, MLT1F1, MER44B, LTR67B, LTR16C, LTR50
